# Care and functional disabilities in daily activities – ELSI-Brazil

**DOI:** 10.11606/S1518-8787.2018052000650

**Published:** 2018-10-25

**Authors:** Karla Cristina Giacomin, Yeda Aparecida Oliveira Duarte, Ana Amélia Camarano, Daniella Pires Nunes, Daniele Fernandes

**Affiliations:** I Secretaria Municipal de Saúde. Diretoria de Assistência. Belo Horizonte, MG, Brasil; II Fundação Oswaldo Cruz. Instituto René Rachou. Núcleo de Estudos em Saúde Pública e Envelhecimento. Belo Horizonte, MG, Brasil; III Universidade de São Paulo. Faculdade de Saúde Pública. São Paulo, SP, Brasil; IV Universidade de São Paulo. Escola de Enfermagem. São Paulo, SP, Brasil; V Instituto de Pesquisa Econômica Aplicada. Diretoria de Estudos e Políticas Sociais. Rio de Janeiro, RJ, Brasil; VI Fundação Getúlio Vargas. Escola Brasileira de Administração Pública e de Empresas. Rio de Janeiro, RJ, Brasil; VII Universidade Federal do Tocantins. Curso de Enfermagem. Palmas, TO, Brasil

**Keywords:** Aged, Aging, Activities of Daily Living, Caregivers, Health Surveys, Idoso, Envelhecimento, Atividades Cotidianas, Cuidadores, Inquéritos Epidemiológicos

## Abstract

**OBJECTIVE:**

To investigate the prevalence of demand and provision of care for the Brazilian population with functional disabilities in activities of daily living.

**METHODS:**

This is a quantitative and descriptive study using baseline data from ELSI-Brazil (Brazilian Longitudinal Study of Aging), a cohort study with a representative sample of the Brazilian population aged 50 years or older (n = 9,412). We considered the demand for care from the self-report of having some difficulty to perform at least one activity of daily life (eating, bathing, going to the toilet, dressing, moving in a room [ambulation], and transferring from chair [transfer]). Care supply was measured by having some help to perform the activity of daily living.

**RESULTS:**

Approximately a quarter of the individuals evaluated (23.2%) reported difficulty in at least one activity of daily living, especially regarding transfer and dressing. Age, schooling, and number of chronic diseases were significantly associated with the difficulty in activities of daily living. Among those who reported difficulty, 35.1% received help of others and 11.8% did not receive (lack of care). The activities with greater lack of care were bathing (13.3%) and transfer (11.7%), which reveals an undignified survival condition. Care remains a family (94.1%) and female (72.1%) issue; despite the important changes that have taken place in society, there is still a lack of care policies. Of the total caregivers, 25.8% reported stopping working or studying to perform this role and only 9.2% were paid (hired ones or family members).

**CONCLUSIONS:**

The ELSI-Brazil results reveal the expressive care demand of the Brazilian population aged 50 years or older with functional disabilities on activities of daily living and the lack of care policies aimed at this public.

## RESUMO

**OBJETIVO:**

Investigar as prevalências da demanda e da oferta de cuidados à população brasileira com limitações funcionais em atividades básicas da vida diária.

**MÉTODOS:**

Estudo quantitativo e descritivo, que utiliza dados da linha de base do ELSI-Brasil (Estudo Longitudinal de Saúde do Idoso), estudo de coorte com amostra representativa da população brasileira de pessoas a partir de 50 anos (n = 9.412). Considera-se a demanda de cuidados a partir do autorrelato de ter alguma dificuldade para o desempenho de pelo menos uma atividade básica da vida diária (comer, tomar banho, ir ao banheiro, vestir-se, locomover-se em um cômodo e transferir-se da cadeira). A oferta de cuidado foi medida por ter alguma ajuda para realizar a atividade básica da vida diária.

**RESULTADOS:**

Cerca de um quarto dos indivíduos avaliados (23,2%) relatou dificuldade em pelo menos uma atividade básica da vida diária, sobretudo quanto à transferência e vestimenta. A idade, a escolaridade e o número de doenças crônicas associaram-se de forma significativa com a dificuldade nas atividades básicas da vida diária. Entre os que referiam dificuldade, 35,1% contavam com a ajuda de outrem e 11,8% não recebiam nenhuma ajuda (insuficiência de cuidado). As atividades com maior insuficiência de cuidado foram: tomar banho (13,3%) e transferir-se (11,7%), o que revela uma condição indigna de sobrevivência. O cuidado continua sendo uma questão familiar (94,1%) e feminina (72,1%); apesar das importantes mudanças ocorridas na sociedade, ainda há insuficiência de políticas de cuidado voltadas para essa área. Dos cuidadores, 25,8% referiram parar de trabalhar ou estudar para exercer essa função; e apenas 9,2% eram remunerados (contratados ou familiares).

**CONCLUSÕES:**

Os resultados do ELSI-Brasil revelam a expressiva demanda de cuidados pela população brasileira a partir de 50 anos com limitações funcionais em atividades básicas de vida diária e a insuficiência de políticas de cuidados voltadas a esse público.

## INTRODUCTION

Currently, Brazil has 54 million persons aged 50 and over, of which 26.5 million are older adults. This population ages with chronic non-communicable diseases in very unequal social contexts that have an impact on unequal care demands and supply^1^.

Data from the Brazilian National Health Survey (PNS-2013) on the care received show that approximately 30% of the Brazilians aged 60 years and over have some difficulty to perform at least one of 10 selected activities of daily living (ADL). Informal care (unpaid) prevailed (approximately 80%), a small proportion received formal care (approximately 6%), 7% received a combination of informal and formal care, and 6% reported receiving no help^2^. Another study showed that the richer strata were more likely to receive any help to perform ADL. In addition, living with another person was associated with the provision of care (formal or informal) for those with lower educational and socioeconomic levels^3^.

Across the world, several ongoing longitudinal international cohorts have addressed the issue of care provision for persons aged 50 years and over with disabilities^4^ ^,^ ^5^. Although it can be understood as medical services provided by health professionals, long t term care is provided more informally (by family and friends) than formally^6^. This informal care, essential to the health system^7^, can be characterized as a hidden health system^8^.

In order to contribute to the planning and adaptation of care policies in a global perspective, the ELSI-Brazil (Brazilian Longitudinal Study of Aging)^9^ was modeled and harmonized with HRS (Health Retirement Studies) and their counterparts in the different continents^5^. The results will allow the evaluation (and improvement) of the quality of this common, singular, and universal experience of caring for others and oneself, by answering the following question: who are the persons who need or provide care for Brazilians aged 50 and over with difficulties in their daily activities?

This work aimed to investigate the prevalence of demand and provision of care for the Brazilian population with functional disabilities in activities of daily living.

## METHODS

The ELSI-Brazil^a^ is a cohort study with a representative sample of the Brazilian population aged 50 years and over. The baseline survey was conducted between 2015 and 2016. More methodological details are described in Lima-Costa et al.^9^ The final sample of the ELSI-Brazil consisted of 9,412 persons aged 50 years and over living in 70 municipalities of different Brazilian regions.

The profile of the care recipients was evaluated according to the following variables:

- Sociodemographic and health: gender, age (50–59 years, 60–69 years, 70–79 years, 80 years and over), schooling level in complete years (illiterate, 1–3, 4–7, 8–11, 12 or more), number of residents in the household, and number of self-reported chronic diseases. The chronic diseases investigated were: hypertension, diabetes, chronic obstructive pulmonary disease, cancer, heart disease (angina, heart failure, or acute myocardial infarction), cerebrovascular accident, arthritis or rheumatism, and osteoporosis.
- Difficulty in performing activities of daily living (ADL): each participant answered separate questions about six activities and their degree of difficulty in performing them. The activities evaluated were: eating, bathing, going to the toilet, dressing, ambulation, and transfer. The ambulation variable was evaluated by the difficulty of the interviewee to walk from one room to another on the same floor, while the transfer activity referred to the act of sitting or getting up from a chair, including wheelchair.

We considered as having difficulty in performing the activities when the participant reported little difficulty, great difficulty, or that they could not perform it. Functional disability was attributed to those who reported some degree of difficulty in performing at least one of these activities.

Regarding the receipt of help, the interviewee was classified as: no help needed, when reporting difficulty in any activity but no need for help; receipt of help, when reporting getting any help to perform any activity ; no receipt of help, when reporting no receipt of help in any activity.

- Need for caregiver: it was asked to those participants who reported difficulty in at least one activity if they had any help. The answers were categorized as: (1) yes, (2) no, if they needed help, and (3) no, because they did not need help. It was also asked who provided the help for each task to those who answered yes.

It was asked to the interviewees the characteristics of caregivers. This refers to the primary caregiver:

- Sociodemographic: gender, age (continuous and categorical), marital status (married, widow/widowed, separated or divorced, single), schooling level (can read or write a message), degree of kinship with the care recipient, type of caregiver (paid or not).

Since the participant could have received help from more than one person, it was considered separately the activities for which the participant obtained help from unpaid (informal care) and paid (formal care) persons. In relation to kinship, it was considered as family caregiver the family member who cared regardless of the geographic distance in relation to the person cared for, with or without remuneration. Non-family members included hired caregivers, who were not family members, and domestic worker.

- Care training: if the caregiver received specific training and, if so, the number of studied hours.
- Care-related characteristics: the number of weekly days spent with caring and whether the caregiver stopped working or studying for it.

All analyses were performed using the procedures for complex samples of the statistical package Stata, version 13.0, considering individual sample weights and sampling parameters. For the analysis, it was used the chi-square test with Rao-Scott correction. Poisson regression analysis was used for the estimates of prevalence ratio and respective 95% confidence intervals for the association between difficulty in ADL and independent variables. It was included the variables that presented p < 0.20 in the multiple model, and those that presented a value of p < 0.05 were kept in the final model.

The ELSI-Brazil was approved by the Research Ethics Committee of the *Instituto René Rachou* of the Oswaldo Cruz Foundation (Protocol 886.754) and all participants of the study signed the informed consent form.

## RESULTS

The distribution of the sociodemographic characteristics of the 9,412 study participants is presented in Table 1. Most were female (54.0%), older adults (53.4%), had more than four schooling years n (67.2%), and had at least one chronic disease (71.7%). Almost a quarter of the individuals reported difficulty in at least one ADL, and the most prevalent was to transfer and to dress.

**Table 1:** Distribution (%) of study participants (≥ 50 years) according to sociodemographic characteristics, impairment of daily activities. Brazilian Longitudinal Study of Aging (ELSI-Brazil), 2015–2016. (n = 9,412)

Characteristics	Total
Sex	
Female	54.0
Male	46.0
Age (in years)	
50–59	47.6
60–69	29.7
70–79	15.6
80 and over	7.1
Schooling (full years of study)	
Zero	13.3
1 to 3	19.5
4 to 7	40.3
8 to 11	18.6
12 or more	8.3
Number of residents in the household (mean and standard error)	3.1 (0.04)
Number of chronic diseases	
Zero	28.3
One	33.5
Two or more	38.2
Impairment of activities	
Transfer	15.7
Dressing	12.7
Bathing	6.1
Ambulation	5.6
Going to the toilet	4.1
Eating	2.3
Number of impaired activities	
Zero	76.8
One	12.7
Two	4.7
Three	2.1
Four	1.4
Five	1.2
Six	1.1
Difficulty in at least 1 activity of daily living	
No	76.8
Yes	23.2

Total	100.0

The prevalence of difficulty in ADL according to sociodemographic characteristics and impairment of ADL is described in Table 2. Age, schooling years, and number of chronic diseases were significantly associated with difficulty in ADL. Among the oldest adults (80 years and over), the prevalence of this outcome was 45% higher than among the youngest. Prevalence was almost triple among the illiterate in relation to the most educated, and it was more than double among those who reported two or more chronic diseases when compared to those who had one or zero.

**Table 2:** Prevalence and prevalence ratio of the study participants (≥ 50 years) according to sociodemographic characteristics and impairment of activities of daily living (ADL). Brazilian Longitudinal Study of Aging (ELSI-Brazil), 2015–2016. (n = 9,412)

Characteristics	Difficulties in ADL		Adjusted PR	95%CI	p

	No	Yes			
Sex					
Male	79.4	20.6	1.00		
Female	74.6	25.4	1.05	0.96–1.14	0.246
Age (in years)					
50–59	80.0	20.0	1.00		
60–69	78.2	21.8	0.89	0.78–1.01	0.090
70–79	73.3	26.7	0.96	0.83–1.10	0.548
80 and over	57.3	42.7	1.45	1.28–1.66	**< 0.001**
Schooling (full years of study)					
Zero	65.4	34.6	2.95	2.30–3.79	**< 0.001**
1 to 3	72.3	27.7	2.45	1.93–3.10	**< 0.001**
4 to 7	76.8	23.2	2.17	1.71–2.75	**< 0.001**
8 to 11	84.2	15.8	1.54	1.19–2.01	**0.001**
12 or more	90.0	10.0	1.00		
Number of residents in the household (mean and standard error)	3.1 (0.04)	3.2 (0.05)	1.02 (0.04)	0.99–1.14	0.060
Number of chronic diseases					
0 to 1	84.9	15.1	1.00		
2 or more	63.6	36.4	2.29	2.11–2.51	**< 0.001**

Total	76.8	23.2			

Values with statistical significance are presented in bold.

Among the participants who reported some difficulties in the performance of ADL, it was verified whether they received the necessary help (35.1%) or if they reported a lack of care (11.8%). It was found the greatest proportions of lack of care among the women, in those individuals aged 60-69 years, with from one to three full schooling years, living in a larger family, and in those with two or more chronic diseases (Table 3).

**Table 3:** Distribution (%) of the study participants (≥ 50 years) with difficulty in activities of daily living (ADL) according to sociodemographic characteristics and receipt of help. Brazilian Longitudinal Study of Aging (ELSI-Brazil), 2015–2016. (n = 2,282)

Sociodemographic characteristics	Receipt of help			p

	No need	Yes	No	
Sex				< 0.001
Male	61.8	32.6	5.6	
Female	47.1	36.9	16.0	
Age (in years)				< 0.001
50–59	56.2	32.3	11.5	
60–69	59.1	27.8	13.1	
70–79	50.5	38.3	11.2	
80 and over	33.9	55.8	10.3	
Schooling (full years of study)				0.002
Zero	43.3	45.7	11.0	
1 to 3	53.8	32.5	13.7	
4 to 7	55.1	33.9	11.0	
8 to 11	60.0	28.4	11.6	
12 or more	60.9	26.0	13.1	
Number of residents in the household (mean and standard error)	3.1 (0.07)	2.5 (0.1)	3.5 (0.07)	0.045
Number of chronic diseases				< 0.001
0 to 1	59.8	30.2	10.0	
2 or more	48.6	38.5	12.9	

Impairment of ADL	53.1	35.1	11.8	

The Figure shows the distribution of the participants with impairment in the performance of some ADL and the receipt or not of care. The receipt of help ranged among activities. The lack of care was greater among persons who reported difficulty in bathing and transferring.

**Figure:**
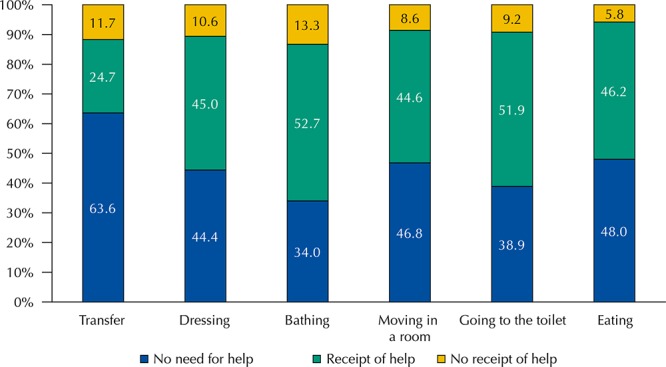
Distribution (%) of the study participants (≥ 50 years) with difficulty in activities of daily living, according to impaired activities and receipt of help. Brazilian Longitudinal Study of Aging (ELSI-Brazil), 2015–2016. (n = 2,282)

Among the persons who identified a primary caregiver, most were cared for by a woman (72.1%). The mean age of caregivers was 48 years; most of them were married, a family member, unpaid, and did not receive care training (Table 4). Among the caregivers, most men provided care to women, while only 53.1% of women cared for other women. Regarding marital status, most female caregivers (61.2%) were married.

**Table 4:** Distribution (%) of primary caregivers according to sociodemographic and care characteristics. Brazilian Longitudinal Study of Aging (ELSI-Brazil), 2015–2016. (n = 828*)

Primary caregiver	Total	Women	Men	p
Sociodemographic characteristics				

Sex	100.0	72.1	27.9	**< 0.001**
Mean age (standard error) in years	48.1 (0.9)	47.4 (0.9)	50.1 (1.8)	0.146
Marital status				0.052
Married	61.4	61.2	61.9	
Single	29.2	27.6	33.4	
Separated/Divorced	6.1	6.8	4.3	
Widow/Widowed	3.3	4.4	0.4	
Education level (can read and write a message)	86.4	86.7	85.6	0.692

Characteristics related to care				

Sex of the care recipient				**< 0.001**
Male	37.9	46.9	14.6	
Female	62.1	53.1	85.4	
Type of caregiver, according to kinship				0.382
Family	94.1	93.5	95.5	
Not family	5.9	6.5	4.5	
Type of caregiver, according to remuneration				**0.004**
Informal	90.8	88.7	96.4	
Formal	9.2	11.3	3.6	
Stopped working/studying to be able to care (yes)	25.8	30.2	14.5	**< 0.001**
Received specific training as caregiver (yes)	6.1	6.4	5.3	0.654
Training received - mean (standard error) in hours	17.1 (4.3)	20.6 (5.0)	5.7 (2.3)	**0.014**
Number of weekly days used for the care of the interviewee				**< 0.001**
Everyday	77.8	81.8	67.2	
Every day except weekends and holidays	1.9	2.5	0.4	
Most days of the week	8.5	7.6	10.8	
At least one day per week	11.8	8.1	21.6	

Total	100.0	72.1	27.9	

* Two caregivers did not present data.

Values with statistical significance are presented in bold.

Regarding to the age group of the caregivers, it was highlighted the presence of older adults (26.1%), of which 57.3% were women and 2.3% were older than 80 years. It was also observed that 6.9% (n = 58) of the caregivers were under 18 years of age, 15.9% of whom were children aged between six and 12 years (data not shown in the Table).

A quarter of the caregivers reported to have left work or school to perform this role. Most caregivers (77.8%) reported working as caregivers the whole week. Only 6.1% of them reported having received some care training. They did it for 17.1 (SD = 4.3) hours on average (Table 4).

## DISCUSSION

Care refers to a set of specific activities combined in a complex life-sustaining network that involves self-care, caring for others, the caregiver, and the care recipient^10^. It usually occurs in domestic spaces, being unpaid^11^. In many countries, the responsibilities for the informal care of fragile family members (older adults, children, the sick, and persons with disabilities) are disproportionately distributed among certain social groups, such as middle-aged women^12^.

It was estimated with ELSI-Brazil data in approximately 12.5 million persons the number of individuals aged 50 years or older that had some difficulty in at least one ADL. In addition, these data confirm that the demand for care is higher among very old persons, those less educated, and women.

However, the increased need for care by the Brazilian aged population occurs together with changes in the social role of women, with their increased participation in labor market^10^ ^,^ ^11^ ^,^ ^13^. In addition, in relation to gender equality, there is a remarkable distance between what is intended (sharing of the responsibility to support the family and taking care of its members) and what is done^1^ ^,^ ^14^, which may further compromise the quality of the care.

In one of the few studies conducted in Brazil that have investigated informal and paid care, Lima-Costa et al.^2^ have estimated that, among the older adults who received help to perform at least one of the 10 selected activities, men were less likely to receive any type of care ( informal, paid, or mixed), regardless of other demographic characteristics. However, according to data of the ELSI-Brazil, although they require less care, men receive more help than women – which may reflect a macho behavior in the Brazilian society^1^ –, as almost twice as many women had to stop working or studying for the care compared to men.

These different views on long-term care, in addition to gender stereotypes, permeate institutional and political factors resulting in great variation in the situations of care for men and women in different cultures^10–14^. Unpaid work – as in the case of care provided in the domestic environment – remains in a gray area^7^ ^,^ ^10^ ^,^ ^15^ while paid work, considered economically productive, is a privileged category of economic and social analysis^13^.

Data from the European Social Survey (2014) show that 34.3% of the adult population worked as informal caregivers in 20 European countries and 7.6% of the caregivers were considered as intensive, since they worked at least 20 hours per week^14^. Although it was not possible to measure the number of hours/day with the ELSI-Brazil data, almost 80% of the caregivers reported working the whole week or most days of the week.

The family responsibility for the care of dependent members assumes that caregivers, especially women, do not have financial or emotional costs when providing care^10^ ^,^ ^13^ ^,^ ^15^ ^,^ ^16^. However, despite the great and undeniable benefits both in the public and private contexts, continuously taking care of someone generates time and money costs and causes loss of opportunities, especially in the labor market, and creates health risks, social isolation, and discouragement of reproduction, among other factors.

In addition, despite the recognized need for some support for the family caregiver ^10^ ^,^ ^14^ ^,^ ^16^ ^-^ ^18^, the formal support network is still very restricted. In Europe, care was more prevalent among women aged 50–59, unemployed ones, especially those who do household chores, and religious persons^19^. In Brazil, although domestic workers – lay workers and without a specific care qualification – have historically also taken care of dependent family members^15^, the participation of this category was quite unimpressive in the studied population. The number of training hours received is noteworthy – it is much less than anticipated by any orientation or training course for caregivers^20^. In addition, for the first time, the hired caregiver appears as the third option among care agents, which requires the understanding of that this work needs to be transformed into a qualified profession to contribute to the care quality.

The act of caring is a stressful experience that can affect the physical and mental health of the caregiver^18^, despite their health conditions^12^ ^,^ ^13^ ^,^ ^16^ and their moment at the course of life. The relationship of the caregiver with a dependent person reduces their chances of keeping a healthy life, deprives them of social contacts, compromises their physical and psychological well-being^17^ ^,^ ^18^, and also exposes them to long-term care (LTC).

In 2015, an International Labor Organization study on LTC coverage for older adults in 46 countries pointed out that informal care for dependent older adults is very common in most evaluated countries. It also points out that this model is not sustainable, mainly because potential informal caregivers are also aging^21^. The “Innovating care for people with multiple chronic conditions” (ICARE4EU) survey has assessed the characteristics of various types of integrated care providers and services in 28 countries of the European Union, Iceland, Switzerland, and Norway. Of the 112 practices investigated, 26.8% included informal caregivers, who worked as co-care providers, and 19.6% included such caregivers as care co-clients. The main elements of the person-centered care are the active participation of patients – in the definition of the care they will receive, the decision making about the care provided, and the self-care –, the involvement of informal caregivers, and the provision of coordinated multidisciplinary care^22^.

In Latin America and Brazil, however, the responsibility for looking after dependent persons is placed primarily on the family. The Government has a subsidiary role: its responsibility is only with olders without family and those economically deprived. This excludes provision of care to those in the middle classes ^23^. However, this model also does not take into account a crucial problem: what is the real capability of the families in providing the care needed?

This scenario exposes two groups that are potentially vulnerable to social security: persons who are dependent for self-care, as they are likely out of productive life, and family members who are not paid to be caregivers as a third of these persons stopped working or studying for this objective. As a result, family caregivers will remain unqualified for the labor market, will not be able to contribute to social security, will have impaired their professional life, and will not have any type of support to continue caring for others. This unfavorable context compromises the future care of these persons.

At the same time, the proportion of non-older adults who demand help with self-care is threatening. When this middle generation requires care, older or younger individuals need to provide it. Despite this, there are persons who currently demand care and are not getting the proper help.

Despite the fact that persons are living longer and enjoying better health conditions, the International Labor Organization (2015) estimates that the LTC needs of older adults are unknown in most of the 46 countries investigated. For most of these countries, the ILO has set a relative threshold of USD 1.461,80 purchasing power parity for the LTC of older adults. According to the report, in Brazil, among the indicators investigated in relation to the percentage of the population aged 65 years and over, the deficit in the legal coverage of LTC was 100%. The public spending on long-term care facilities for older adults per person aged 65 and over, as a percentage of GDP per capita in 2013, was 0 (zero); public spending on LTC, as a percentage of GDP, in the 2006-2010 average was 0 (zero); the difference between coverage and uncovered percentage of the population aged 65 years and over because of a lack of financial resources was 100%; the number of formal workers of LTC per 100 persons aged 65 and over was 0 (zero). It is estimated that the coverage gap, that is, the percentage of the population not covered because of the lack of formal LTC workers (relative threshold: 4.2 workers per 100 persons aged 65 years and over), would be 100%. Thus, the country would require more than 650,000 formal caregivers to fill the gap of LTC. However, only the offer of formal caregivers does not set up a LTC policy^19^.

According to the World Health Organization (WHO)^24^, “thinking about a LTC policy for the Brazilian older population means thinking, first and foremost, about ensuring the access of all older citizens to such care as a social right” (p. 343). Considering the results of this study, the reflection on future perspectives for Brazil^25^ means building care policies for persons of all ages. Camarano asks: “would this be a new social risk to be taken”^13^?

The WHO projections point to a 400% increase in demand for LTC for the older population living in developing countries, including Brazil^24^. It is estimated with ELSI-Brazil data that the lack of proper care reaches approximately 1.5 million persons among those aged 50 and over.

Caring for a growing population is a challenge and a necessity for which the country has not prepared itself. To face the reality in place, the country needs to be aware of this situation and run against time^25^. Nevertheless, in Brazil, the debate around LTC finds resistance, not only because of its proximity to subjects considered taboo, such as disease, disability, frailty, old age, and death, but also because of contradictions between models and conceptions about the roles that the Government should have as a provider of welfare for its population^26^, especially in times of political and fiscal crisis.

The results of this study are important because it is the first Brazilian population-based study to reveal the dyad involved in the care of persons with ADL dependency, the real demands, and the gaps in the care provision. However, there are limitations: the cross-sectional design of the study allows us to demonstrate an association between the phenomena studied, but no causal relationship can be inferred. Another limitation is the fact that the data are based exclusively on the self-report of the interviewees, which can be influenced by environmental, physical, social, and psycho-emotional factors. The continuation of the longitudinal research may better determine the behavior of the care demand and supply in the Brazilian population aged 50 years and over.

We must reflect on the excessive confidence that the Brazilian Government places on the family caregiver while ignoring the physical, emotional, and financial burden that the permanent care of persons in situations of dependency imposes. It is expected a reduction in the supply of family caregivers. There is an urgent need to place this matter on the agenda of Brazilian public policies, recognizing it as the fourth pillar of social security^26^.

In the perspective of a growing crisis of what is termed “family insufficiency,” the answer should be the recognition of care as part of the human condition. This will require the involvement of the family, government, and society in the broader understanding that successful aging will not be able to exclude the possibility of disability and need for care^27^. That is why, in our view, it would not be correct to speak of family insufficiency, but rather of insufficient care policies that support families in their need to care for themselves. The LTC must be effectively materialized through the strengthening of the Brazilian Unified Health System (SUS) and the Unified Social Assistance System (SUAS), as well as the compliance with the various constitutional and infraconstitutional rules that ensure the right to care for all Brazilians.

The support of the caring family means understanding care as a transverse dimension of the health and well-being of the population, which requires the sharing of tasks between genders and generations and dignity in the care offered to persons of all ages.

## CONCLUSIONS

In Brazil, similar to what occurs in most countries – including those that have always assigned to the family the main role in the care of older adults, such as Japan –, the greater demand for care is followed by the decreased potential supply of family caregivers given the changes in family profile and the role of women. Women are the main caregivers and at the same time the most vulnerable among the segments that demand care.

Therefore, it is up to the Brazilian Government to consider the demographic reality and be the driving force capable of providing an intersectoral and integrated LTC policy, and to anticipate the care needs that extrapolate health ones. This includes regulating the caregiver profession and protecting the family caregiver and care recipient, as well as developing strategies for the prevention and recovery of disability and frailty over the life course.

## Footnotes

a Fundação Oswaldo Cruz. Brazilian Longitudinal Study of Aging. Rio de Janeiro; c2015 [cited 2017 Nov 28]. Available from: http://elsi.cpqrr.fiocruz.br
